# Association of Obesity and Surgery Outcomes in Patients with Endometrial Cancer: A Single-Center Analysis

**DOI:** 10.1055/s-0042-1759632

**Published:** 2022-12-29

**Authors:** Savas Ozdemir, Gul Ozel Dogan

**Affiliations:** 1 Department of Gynecology and Obstetrics, University of Health Sciences, Prof. Dr. Cemil Tascioglu City Hospital, Istanbul, Turkey

**Keywords:** BMI, endometrial cancer, obesity, surgery

## Abstract

**Objective** Although obesity can result in high morbidity and mortality in surgical outcomes because of multiple comorbidities, determinants of outcome in obese patients who underwent endometrial cancer surgery remain unclear. The aim of this study is to assess the relationship between body mass index (BMI) and surgical outcomes in obese patients with endometrial cancer.

**Methods** An institutional retrospective review of the demographic details, clinical characteristics, and follow-up data of 142 patients with endometrial cancer who underwent surgery during a 72-month period was performed. The patients were divided into three groups based on their BMI; patients with BMI < 25 were identified as normal weight, patients with BMI between 25 and 30 were accepted as overweight, and those with BMI ≥ 30 kg/m ^2^ were identified as obese. The groups' demographic and clinical variables were compared.

**Results** Of the 142 patients, 42 were in the normal weight group, 55 in the overweight group, and 45 in the obese group. Age, surgical procedures, blood loss, preoperative health status, and metastatic lymph nodes did not show a significant difference between groups. However, surgery time and total lymph nodes were higher in the obese group. ( *p* = 0.02, *p* = 0.00, and *p* = 0.00, respectively). Common complications were anemia, fever, intestinal injury, deep vein thrombosis, fascial dehiscence and urinary infection. There was no significant difference according to the complications.

**Conclusion** Our results indicated that higher BMI was significantly associated with a longer duration of endometrial cancer surgery. Minimally invasive surgeries and conventional laparotomy could be performed safely in obese patients.

## Introduction

Obesity is a well-established risk factor for developing endometrial cancer, more than any other cancer type. 1 Insulin resistance is responsible for releasing growth factors for cellular proliferation, higher levels of interleukins, tumor necrosis factors, and adipokines causing an obesity-related proinflammatory state, and high estrogen levels through increased aromatase activity in adipose tissue are proposed as contributors to the increased risk of developing endometrial cancer. 2 Endometrial cancer is known as a hormone-dependent type of cancer. Obesity affects hormone metabolism by increasing the aromatization of androstenedione to estrone in adipose tissues and causes an increase in the circulating levels of estrogen, creating a favorable environment for tumor formation. 3 The incidence of endometrial cancer is projected to rise as women's obesity rates continue to rise. In a study by Ward et al. 4 with 33,232 endometrial cancer patients, it was reported that the 10-year mortality due to endometrial cancer was associated with death due to cardiovascular disease. It was the most common reason related to morbid obesity. Obesity and endometrial cancer have been linked in numerous research studies. 5 6 7 8 Although excess body fat is a significant risk factor for endometrial cancer, its impact on survival is unclear.

Surgical procedures for treating endometrial cancer are hysterectomy, bilateral salpingo-oophorectomy, and pelvic and para-aortic lymphadenectomy. Many studies have established that obese patients are at a higher risk of perioperative and postoperative complications, such as longer hospital stay and increased morbidity, even when minimally invasive surgeries or laparotomy are performed. 9 10 11 Obesity is defined by the World Health Organization (WHO) by using the body mass index (BMI) cutoff point of > 30 kg/m ^2^ , which is calculated as weight in kg divided by height in meters squared. Body mass index has significant public health importance because it correlates well with morbidity and/or mortality and endometrial cancer risk. Although surgery is the standard procedure in the staging and treatment of endometrial cancer, obesity may affect surgical outcomes due to its accompanying comorbid disorders. The impact of morbid obesity on endometrial cancer patients' survival is crucial as postoperative complications among obese women seem to be higher than among their normal-weight counterparts. 12

The evaluation of the effects of obesity on surgical outcomes may help decrease morbidity and improve prognosis in patients with endometrial cancer. However, there are insufficient data in the current literature that explain the impact of morbid obesity on the surgical outcomes of endometrial cancer and compare it with the endometrial cancer patients with normal weight. This study aims to determine the link between BMI and surgical outcomes in obese endometrial cancer patients.

## Methods

### Participants

This study was performed in accordance with the ethical standards of the Helsinki declaration. Ethical approval was obtained for this retrospective, cross-sectional study by the ethics committee of the University of Health Sciences, Sisli Hamidiye Etfal Education and Research Hospital. Informed consent was obtained routinely. We reviewed the records of patients older than 18 years with endometrial cancer admitted to our hospital's gynecologic oncology department within a 72-month period from 2014 to 2020.

### Inclusion and Exclusion Criteria

The inclusion criteria are listed below:

1. Patients with pathologically proven endometrial cancer.
2. Patients older than 18 years.
3. A detailed medical record including patient's history, clinical findings, laboratory and pathology test results, treatment outcomes, etc.

The exclusion criteria are as follows:

1. Patients without a definite pathologic diagnosis
2. Patients with secondary cancer.
3. Patients with endometrial cancer who were treated conservatively.

### Data Collection

The patients' demographic characteristics (age, sex), weight, height and body mass index (BMI), surgical procedure (total hysterectomy-bilateral salpingo-oophorectomy [via laparotomy or laparoscopy] with or without pelvic and para-aortic lymphadenectomy), duration of hospital stay, lymph node involvement, the average number of lymph nodes removed, routine biochemical examination, preoperative evaluation and preparation for anesthesia, perioperative and postoperative complications, and follow-up data were recorded. The patients were divided into three groups based on their BMI. Body mass index [kg/height (m)]2 was calculated and classified according to the World Health Organization (WHO) guidelines. Thus, patients with BMI < 25 were identified as normal weight, patients with BMI from 25 to 30 were accepted as overweight, and those with BMI ≥ 30 kg/m ^2^ were identified as obese. Later, we compared the variables mentioned above and surgical outcomes according to patients' BMI.

### Statistical Analysis

Data were analyzed using the IBM SPSS Statistics for Windows, version 23.0 software (IBM Corp., Armonk, NY, USA). Descriptive statistics (mean, standard deviation, frequency, and percentage) were used for the demographic and clinical characteristics. The analysis of variance (ANOVA) test followed by Tukey multiple comparison methods among these three BMI groups was performed. The categorical variables were compared using the Chi-squared test. A *p* -value of < 0.05 was considered to be statistically significant.

## Results

During the 6-year period, 142 patients were operated on for endometrial cancer. These surgical procedures were performed via laparotomy or conventional laparoscopy, which were performed at our hospital's gynecologic oncology unit. The mean age of the subjects was 60.52 ± 9.89 years (range, 18–82 years). The in-hospital mortality rate was 0%. The number of patients with BMI < 25 (normal weight) was 42 (29.6%), those with BMI from 25 to 30 (overweight) were 55 (38.7%), and patients with BMI > 30 (obese) were 45 (31.7%).

The demographic and clinical characteristics of patients with a comparison between the three groups are demonstrated in Table 1 . There was no significant difference between the groups regarding age, surgical procedures, presence of menopause, intraoperative bleeding, preoperative health status (ASA), the mean duration of hospital stay, CA125 level, and the number of metastatic nodes. However, duration of surgery, the number of total nodes and non-metastatic nodes differed significantly and were higher in the group of patients with BMI > 30 ( *p* = 0.02, *p* = 0.00, and *p* = 0.00, respectively). Table 2 shows the posthoc Tukey test results of these variables.

**Table 1:** Demographic and clinical characteristics of patient groups

Variables	BMI < 25 (normal weight)	BMI from 25 to 30 (overweight)	BMI > 30 (obese)	*P* -value
Number of patients	42 (29.6%)	55 (38.7%)	45 (31.7%)	−
Age	61.00 ± 11.15	60.00 ± 10.56	60.44 ± 10.42	0.901
Surgical procedure				
* Laparotomy*	25 (17.6%)	31 (21.8%)	26 (18.3%)	0.952
* Laparoscopy*	17 (12.0%)	24 (16.9%)	19 (13.4%)	
Menopause				
* Premenopause*	7 (4.9%)	10 (7.0%)	9 (6.3%)	0.922
* Postmenopause*	35 (24.6%)	45 (31.7%)	36 (25.4%)	
Estimated blood loss (ml)	253.81 ± 89.25	263.64 ± 108.77	273.33 ± 111.88	0.685
Surgery time (minutes)	142.14 ± 25.50	158.27 ± 36.69	169.89 ± 44.18	0.002
ASA	1.62 ± 0.66	1.49 ± 0.57	1.47 ± 0.59	0.451
Duration of hospital stay (days)	5.64 ± 1.91	5.16 ± 1.75	5.56 ± 2.03	0.404
Pre-CA125	23.98 ± 34.58	48.58 ± 139.33	54.16 ± 132.77	0.436
Number of total dissected pelvic lymph nodes	19.10 ± 10.62	23.47 ± 11.74	34.84 ± 14.31	0.000
Metastatic nodes	1.21 ± 2.08	1.05 ± 2.38	0.93 ± 2.06	0.837
Non-metastatic nodes	18.00 ± 10.76	22.42 ± 12.03	33.91 ± 13.90	0.000

**Table 2:** The posthoc comparisons using Tukey's HSD

Factor	Pairwise Comparison	*P* -value
Surgery time (minutes)	Normal weight vs Overweight	0.083
	Overweight vs Obese	0.256
	Normal weight vs Obese	0.002
Total nodes	Normal weight vs Overweight	0.196
	Overweight vs Obese	0.000
	Normal weight vs Obese	0.000
Non-metastatic	Normal vs Overweighted	0.190
	Overweight vs Obese	0.000
	Normal weight vs Obese	0.000

Patient complaints at the time of admission and complications to our outpatient clinic according to patient groups were summarized in Table 3 . The reasons for patients' admissions were vaginal bleeding, abdominal pain, itching, and routine examination. The symptoms for hospital admission did not differ according to patients' BMI. Anemia (5.6%), fever (1.4%), intestinal injury (0.7%), deep vein thrombosis (0.7%), fascial dehiscence (0.7%), rupture of veins (0.7%), surgical site infections (0.7%), and urinary infection (2.8%) were common perioperative (intraoperative and postoperative) complications among all patients. There was no significant difference between the groups according to the perioperative complications.

**Table 3:** Perioperative data and complications according to patient groups

	Clinical features	BMI < 25 (normal weight)	BMI from 25–30 (overweight)	BMI > 30 (obese)	Total (n)
Symptoms for hospital admission	Bleeding	37 (26.1%)	38 (26.8%)	31 (21.8%)	106 (74.6%)
	Abdominal pain	0 (0.0%)	1 (0.7%)	1 (0.7%)	2 (1.4%)	
	Itching	0 (0.0%)	1 (0.7%)	1 (0.7%)	2 (1.4%)	
	Routine examination	5 (3.5%)	15 (10.6%)	12 (8.5%)	32 (22.5%)	
Perioperative complications	Atrial fibrillation	1 (0.7%)	0 (0.0%)	0 (0.0%)	1 (0.7%)
	Anemia	3 (2.1%)	2 (1.4%)	3 (2.1%)	8 (5.6%)	
	Fever	0 (0.0%)	1 (0.7%)	1 (0.7%)	2 (1.4%)	
	Intestinal injury	0 (0.0%)	0 (0.0%)	1 (0.7%)	1 (0.7%)	
	Deep vein thrombosis	0 (0.0%)	0 (0.0%)	1 (0.7%)	1 (0.7%)	
	Fascial dehiscence	0 (0.0%)	0 (0.0%)	1 (0.7%)	1 (0.7%)	
	Rupture of the iliac vein	0 (0.0%)	1 (0.7%)	0 (0.0%)	1 (0.7%)	
	Bleeding of obturator vein	0 (0.0%)	1 (0.7%)	0 (0.0%)	1 (0.7%)	
	Urinary infection	1 (0.7%)	2 (1.4%)	1 (0.7%)	4 (2.8%)	
	Surgical site infections	0 (0.0%)	1 (0.7%)	0 (0.0%)	1 (0.7%)	
	None	37 (26.1%)	47 (33.1%)	37 (26.1%)	121 (85.2%)	

## Discussion

The current study aimed to demonstrate the surgical outcomes pertaining to the management of endometrial cancer patients according to their BMI and to explore which variables were significant in patient morbidity. Our statistical analysis demonstrated that duration of surgery, number of total dissected pelvic lymph nodes, and non-metastatic nodes were higher in endometrial cancer patients with obesity. Demographic, clinical, and laboratory findings, such as patient's age, presence of menopause, blood loss, preoperative health status, hospital stay, and CA125 levels, were not related to patients' BMI. Moreover, our results indicate that surgical procedures which were performed via laparotomy or laparoscopy had no significant relationship with the subjects' weight. The symptoms for hospital admission did not differ according to patients' BMI. In addition, perioperative complications did not significantly differ among patient groups.

Kokts-Porietis et al. 13 reviewed the studies that included estimated body fat with BMI to evaluate the relationship between obesity and mortality among endometrial cancer survivors. They reported that endometrial cancer survivors who were obese at the time of diagnosis had a higher risk of cancer recurrence and all-cause death but not endometrial cancer-specific mortality. In our study sample, the in-hospital mortality rate was also 0%, and we suggest that elevated BMI might not be related to mortality in endometrial cancer.

In a retrospective study by Ward et al., 4 the authors evaluated the causes of death among women with endometrial cancer. They found that cardiovascular diseases were the leading reason of death from endometrial cancer. Although the causes of death in patients with endometrial cancer were not the main concern of the current study, we found that the patients with BMI > 30 had a lower preoperative health status, which shows the preoperative chronic medical conditions.

In a review by Onstad et al., 14 the authors suggested that operating on obese patients was more difficult than on normal-weight patients with endometrial cancer due to technical aspects of the surgery that could affect visualization. In the current study, we detected that the duration of surgery in the obese patient group was longer than in the normal-weighted and overweight patients, which supports the researchers' conclusion. However, obesity did not significantly impact the surgical procedures in the three groups of this study. Similar to our results, Gabala et al. 10 reported that obesity did not affect the surgical techniques in endometrial cancer. In a study by Erkanli et al., 15 they investigated the effect of BMI on clinical and pathologic features and surgical morbidity in 42 patients with endometrial cancer. The number of participants was higher in the current study, and our findings support their results. They also did not find any difference in length of hospital stay and intraoperative or postoperative complications. It can be concluded that the surgical approach might be performed safely in morbidly obese endometrial cancer patients.

There are mixed results in the retrospective studies regarding the impact of obesity on operative complications in the current literature. Similar to previous studies, we detected that postoperative complication rates did not differ significantly between the obese and non-obese patient groups. 10 11 15 16 On the contrary, Bouwman et al. 17 reported that elevated BMI was associated with an increased risk of postoperative surgical complications in morbidly obese patients who underwent laparotomy. Patients' characteristics may explain these different results, and they also depend on surgeons' experience and type of equipment. Only 14.8% of our patient sample had suffered from perioperative complications. Arrhythmia, blood loss, high fever, intestinal injury, deep vein thrombosis, fascial dehiscence, bleeding of veins, urinary infection and surgical site infections were detected. Even though obesity did not affect the course of surgery in this study, we believe that when considering endometrial cancer surgery, it is critical to recognize these complications to avoid them.

Endometrial cancer continues to increase in incidence and mortality. Obesity is now recognized as an independent risk factor for endometrial cancer, accounting for more than half of all cases. Women diagnosed with endometrial cancer with a high BMI have a higher probability of morbidity. For this reason, obesity may adversely affect surgery outcomes. Therefore, we think that more studies determining this relationship between endometrial cancer and obesity are needed in the medical literature.

This study has some limitations. First, only eligible data in the record were assessed because of its retrospective nature. Second, the study was performed at a single center. In addition, all the patients were operated on by the same experienced surgeons. However, a long-term period and a relatively high number of participants are the strengths of this study.

## Conclusion

In conclusion, our findings indicate that increased BMI is related to higher surgery time in patients with endometrial cancer. However, obesity did not impact surgical outcomes such as blood loss, duration of hospital stay, and complication rates. Moreover, mortality rates in endometrial cancer were not affected by BMI. Endometrial cancer surgery can be safely and adequately managed among patients with obesity. Further prospective studies evaluating the impact of obesity on long-term follow-up of endometrial cancer surgery are warranted.
